# RNA-seq analysis of *Pichia anomala* reveals important mechanisms required for survival at low pH

**DOI:** 10.1186/s12934-015-0331-4

**Published:** 2015-09-16

**Authors:** Eugene Fletcher, Amir Feizi, SungSoo Kim, Verena Siewers, Jens Nielsen

**Affiliations:** Department of Biology and Biological Engineering, Chalmers University of Technology, Kemivägen 10, 412 96 Gothenburg, Sweden; Samsung Advanced Institute of Technology, 130 Samsung-Ro YoungTong-Ku, Suwon, Kyunggi-do South Korea; Novo Nordisk Foundation Center for Biosustainability, Chalmers University of Technology, Kemivägen 10, 412 96 Gothenburg, Sweden; Novo Nordisk Foundation Center for Biosustainability, Technical University of Denmark, 2970 Hørsholm, Denmark; Biotech Research Team, Dongbu Farm Hannong Co., Ltd., Daejeon, 305-708 Republic of Korea

**Keywords:** *Pichia anomala*, Low pH, Stress, Biochemicals, RNA-seq, Genome sequencing, ATP production

## Abstract

**Background:**

The product yield and titers of biological processes involving the conversion of biomass to desirable chemicals can be limited by environmental stresses encountered by the microbial hosts used for the bioconversion. One of these main stresses is growth inhibition due to exposure to low pH conditions. In order to circumvent this problem, understanding the biological mechanisms involved in acid stress response and tolerance is essential. Characterisation of wild yeasts that have a natural ability to resist such harsh conditions will pave the way to understand the biological basis underlying acid stress resistance. *Pichia anomala* possesses a unique ability to adapt to and tolerate a number of environmental stresses particularly low pH stress giving it the advantage to outcompete other microorganisms under such conditions. However, the genetic basis of this resistance has not been previously studied.

**Results:**

To this end, we isolated an acid resistant strain of *P. anomala*, performed a gross phenotypic characterisation at low pH and also performed a whole genome and total RNA sequencing. By integrating the RNA-seq data with the genome sequencing data, we found that several genes associated with different biological processes including proton efflux, the electron transfer chain and oxidative phosphorylation were highly expressed in *P. anomala* cells grown in low pH media. We therefore present data supporting the notion that a high expression of proton pumps in the plasma membrane coupled with an increase in mitochondrial ATP production enables the high level of acid stress tolerance of *P. anomala*.

**Conclusions:**

Our findings provide insight into the molecular and genetic basis of low pH tolerance in *P. anomala* which was previously unknown. Ultimately, this is a step towards developing non-conventional yeasts such as *P. anomala* for the production of industrially relevant chemicals under low pH conditions.

**Electronic supplementary material:**

The online version of this article (doi:10.1186/s12934-015-0331-4) contains supplementary material, which is available to authorized users.

## Background

The conversion of lignocellulosic feedstocks into next-generation fuels and other industrially relevant chemicals has taken central stage in recent years. This involves several upstream and downstream processes which can be summarized as pre-treatment, saccharification, fermentation and product recovery [1]. Microorganisms are generally used as cell factories for the biological conversion of feedstocks to the final products. In order to reduce costs and improve the efficiency of this process, it is preferable to use micro-organisms that can survive the harsh environment created by the pre-treatment step and also be tolerant to the toxicity of the final product. When using lignocellulosic materials as feedstock, an acid pre-treatment step is often required to effectively breakdown the lignin fibers trapping the carbohydrates that need to be hydrolysed before fermentation [2]. There is also the release of weak acids and other toxic compounds in the process resulting in severe consequences on the growth and performance of the microbial host. Furthermore, in the production of biofuels and organic acids from lignocellulose or other feedstocks, the principal stress encountered by the cell is low pH stress which results in growth inhibition and low productivity. Therefore, not only must the micro-organism used be able to survive the acid pre-treatment stage, but also the low pH conditions required for the production and product recovery.

Fungi have been used in the food industry for several centuries and species such as *Saccharomyces cerevisiae* and *Aspergillus niger* have now been established as the most widely used microbial hosts for the production of biochemicals such as biofuels, organic acids, among others from biomass [3, 4]. Despite the wide use of *S. cerevisiae* in industrial fermentations, its use is restricted by its limited resistance to the different stress conditions encountered under an industrial setting, particularly acid stress.

Currently, strains of *S. cerevisiae* are being engineered to overcome the stress constraints posed by low pH environments typical for an industrial setting [5]. As an alternative approach, however, isolation and utilization of microbes that can survive under these harsh acidic conditions can prove useful. Furthermore, studying the biological mechanisms behind the tolerance of superior yeasts will be important in revealing novel stress resistance mechanisms. Therefore, these strains can be developed as novel cell factories since they already possess the tolerance phenotype. One example is *Pichia anomala* (also known as *Wickerhamomyces anomalus* or *Hansenula anomala*), a yeast that is widespread in nature and has been isolated from different environments including the skin of fruits and contaminated wine [6]. *Pichia anomala* is a robust yeast and possesses innate stress adaptation mechanisms making it resistant to a number of environmental stresses and it has also been described as having ‘potential biotechnology applications’ such as in bioremediation, antimicrobial and bioethanol production [6, 7]. Some studies have mentioned *P. anomala’s* resistance to low pH [7, 8]. A number of acid stress response mechanisms have been identified in *S. cerevisiae* and other fungi which include maintenance of the cell wall structure, metal metabolism and proton efflux by the membrane ATPases [9, 10].

While the advent of new sequencing technologies has revolutionized our knowledge on model organisms at the genome scale, these technologies also facilitate the study of novel interesting species, which have potential medical or industrial application. The draft genome of *P. anomala* was recently published and annotated to identify genes that code for proteins with antimicrobial activities [11]. However, to date, the genetic basis of the strain’s resistance to low pH remains elusive as there is no information available on the subject.

Here, we first studied the physiological properties of a strain of *P. anomala*, isolated from low pH cultures. Its identity was confirmed based on the DNA sequence of its Internal Transcribed Sequence (ITS) region. We then, used whole genome sequencing and RNA-seq analysis to identify biological mechanisms that can be linked to the survival of this yeast at low pH.

## Results

### Identification of *P. anomala*

We set up a series of shake flask cultures at low pH. Initially, the cultures were inoculated with *Saccharomyces cerevisiae* CEN.PK113-7D, but during the course of an evolution experiment we identified rapid appearance of five yeast strains that we then isolated from the low pH (pH 3.0) cultures. We amplified by PCR the conserved Internal Transcribed Sequence (ITS) region of the yeasts and that of *S. cerevisiae*. All five isolated strains produced a band of size ~650 bp. These PCR band sizes were compared to that produced by *S. cerevisiae* which had a size of ~840 bp (Additional file 1: Figure S1). A BLASTN (http://www.blast.ncbi.nlm.nih.gov/Blast) search of the sequenced PCR products revealed that the isolated yeast strains were *Wickerhamomyces anomalus* (also known as *P. anomala*) with a 100 % identity and coverage. Thus, we realized that this yeast clearly had a strong competitive advantage at low pH over *S. cerevisiae* and we therefore undertook a detailed physiological characterisation of this yeast.

### Physiological characterisation

**Fig. 1:**
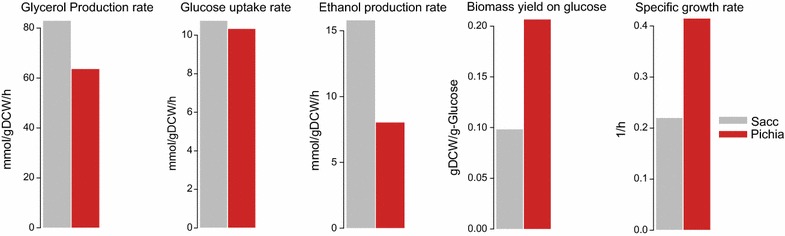
Physiological characterisation of *S. cerevisiae* (*grey bars*) and *P. anomala* (*red bars*) at pH 3.0. *Graphs* show the glycerol production rate, glucose uptake rate, ethanol production rate, biomass yield on glucose and specific growth rate of both strains. The data represents three biological replicates. *DCW* dry cell weight

The five isolated strains were screened in low pH media (pH 3.0) to identify the top- performing strain (i.e. the most resistant of the five strains) which was used in the subsequent experiments. The criterion used for this selection was the differences in growth rate of the isolates when grown at this pH. Even though the difference in growth rate among the five isolates was not pronounced, we selected Isolate 5 as the top-performing strain since it had a slightly higher growth rate compared to the other four isolates (Additional file 2: Figure S2). To determine the effect of low pH on the top-performing *P. anomala* strain isolated, the gross phenotype of this low pH resistant strain in minimal media at pH 3.0 was characterised and this was compared to *S. cerevisiae* (Fig. 1). At this pH, the *P. anomala* strain grew at a rate (0.42 h^−1^) which was two-fold higher than the growth rate of the *S. cerevisiae* strain (0.22 h^−1^). The glucose uptake rates, biomass yield on glucose, ethanol and glycerol production rates were also measured and compared between these two strains under this stress condition. Even though both yeasts took up glucose at an identical rate of 11.2 mol/gDCW/h (*S. cerevisiae*) and 10.8 mol/gDCW/h (*P. anomala*; *p* > 0.5), the biomass yield on glucose was two-fold higher in *P. anomala* (0.23 gDCW per gram of glucose) compared to *S. cerevisiae* (0.11 gDCW per gram of glucose**)**. On the other hand, per dry cell weight, *S. cerevisiae* produced ethanol and glycerol faster than *P. anomala* (Fig. 1). Being a Crabtree negative yeast, it was surprising to observe ethanol production by the *P. anomala* strain. However, the ethanol produced was just minimal and indeed, other studies have reported ethanol production by *P. anomala* [7, 12, 13]. The final pH after shake flask fermentations was measured. The pH dropped from 3.0 to 2.40 and 2.69 in the *P. anomala* and *S. cerevisiae* cultures respectively suggesting a more active secretion of intracellular metabolites by *P. anomala* which acidifies the medium. Growth at this pH also shows the ability of the strain to survive at even lower pH conditions (i.e. pH <2.5).

### Genome sequencing and assembly

The first draft of the *P. anomala* genome was assembled by the U.S. Department of Energy Joint Genome Institute (JGI) using Roche (454) and Illumina data in 2011. In this draft, the *P. anomala* genome size is ~14 Mbp with 6,423 gene models. To further confirm the identity of our isolated strain and to compare its genomic features with the assembled genome by the JGI, we sequenced all five isolated strains using the Illumina platform to generate 100-bp paired-end reads. Two libraries were prepared with mean fragment sizes of 460 and 560 bp respectively and sequenced obtaining 30 millions of fragments for a total of 3.2 Gb, corresponding to approximately 300×-fold coverage of the final assembly. The *P. anomala* genome of our isolated strains was assembled de novo by three different pipelines based on de Bruijn graph-based (DBG) assemblers (see “Methods”) including ABySS [14], SPAdes [15], and Velvet [16]. The assembled genomes were compared with each other using the JGI draft assembly as a reference genome with the QUAST program [17]. The ABySS assembler outperformed the other two assemblers in terms of genome coverage yielding assembled contigs containing 80 % of the genomes (Additional file 3: Table S1). The assembly statistics are summarized in Table 1, comprising 7079 contigs with an N50 of 5923 bp for a total sequence of 54.9 Mb. The BLASTN of the top 10 longest contigs from ABySS output resulted in >98 % identities with the scaffolds of the JGI draft assemblies. Therefore, we could confirm the strains to be *P. anomala*. The JGI assembly draft includes reasonable annotation accessible from its browser (http://www.genome.jgi-psf.org/pages/search-for-genes.jsf?organism=Wican1). To avoid redundancy, we did not annotate our assembled genome and we used the provided annotation. The JGI also provided the possibility to compare the gene catalogs between three fungi—*Pichia stipitis,Hansenula polymorpha* NCYC 495 leu1.1, and *Saccharomyces cerevisiae* S288C in an interactive way. Therefore, we focused on characterisation of our strain’s resistance to low pH.

**Table 1:** *P. anomala* genome statistics

	Contigs	Scaffolds
Number	28,886	25,610
Number of contigs >500 bp	5840	4604
N50	5923	7245
N80	3008	3818
Maximum size	82,187	99,153
Total assembly size	2.41E+07	2.43E+07

	Contigs
Genome fraction (%)	80.313
# predicted genes (unique)	6787
# predicted genes (≥300 bp)	7414
# predicted genes ≥1500 bp)	2724
# predicted genes (≥3000 bp)	676
GC (%)	34.44

### Defining the transcriptome of *Pichia anomala* at low pH

**Fig. 2:**
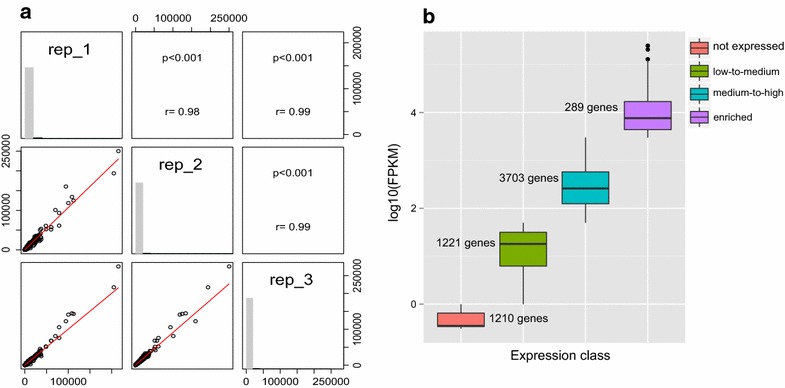
Transcriptome analysis of *P. anomala* at low pH. The RNA-seq data from three biological replicates were analyzed and a correlation plot of the FPKM values was obtained for the replicates (**a**). The normalized FPKM values were calculated using the Tuxedo pipeline [18] and were used to classify the *P. anomala* transcriptome using a *boxplot* of the four gene expression classes (based on cutoff in the Additional file 4: Figure S3). The number of the genes in each class are above each *boxplot* (**b**)

Although JGI provided the first annotated draft for *P. anomala*, there has not been any transcriptome analysis to explore the expression profile of this interesting fungus. Therefore, in order to determine the genes or pathways that respond to low pH in *P. anomala*, we grew the cells in minimal media at pH 3.0 in batch cultures until they reached the mid-exponential phase (OD ~1) and the total RNA was extracted for RNA-seq analysis. The readouts were used as input for the tuxedo pipeline for the estimation of the FPKM (Fragments Per Kilobase of exon per Million fragments mapped) values and corresponding analysis (see “Methods”). The normalized FPKM values from the tuxedo pipeline were used as indication of the gene expression level when *P. anomala* was exposed to low pH (Fig. 2a). To simplify the analysis, we categorized the genes based on their FPKM values into four expression levels including, non-expressed (1210 genes), low to medium expressed (1221 genes), medium to high expressed (3703 genes) and genes with enriched level of expression (289) (Fig. 2b, Additional file 4: Figure S3).

**Fig. 3:**
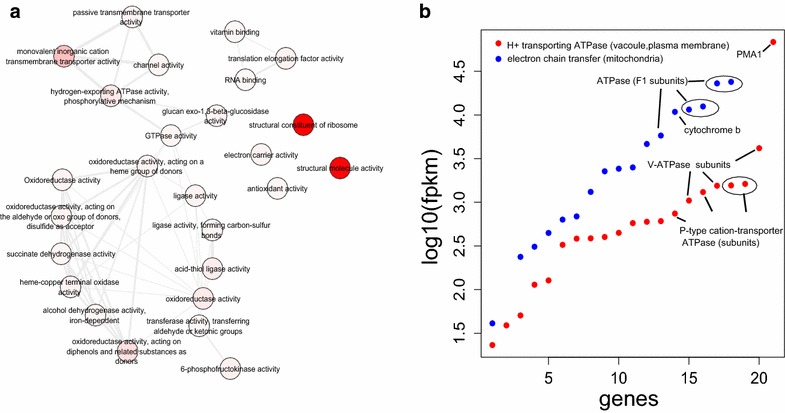
GO enrichment network of the highly expressed genes (enriched category) of the *P. anomala* strain. The GO graph was obtained from REVIGO [19]. The nodes indicate the enrichment terms and highly similar GO terms are linked by edges. The node color reflects the significance of the GO term (**a**). A comparison of the log_10_(FPKM) values and the number of the genes coding proteins involved in the electron transfer chain and H^+^ transport ATPase (**b**)

To detect the target genes potentially responsible for the low pH resistance, we first performed a Gene Ontology (GO) enrichment analysis on all four categorized expression levels (Additional file 5: Figure S4, Additional file 6: Figure S5, Additional file 7: Figure S6). We found GO terms related with low pH resistance in the enriched category with extremely high FPKM values. The most significant GO terms included the genes mapped to hydrogen transport to the vacuole and out into the extracellular environment such as hydrogen ion transmembrane transporter activity (GO:0015078) and hydrogen-exporting ATPase activity (GO:0036442) (Fig. 3a).

### Genes involved in proton efflux (H^+^-ATPases)

In Fig. 3b, the genes that mapped to the hydrogen-exporting ATPase activity GO terms are shown and these consist of the plasma membrane P-type H^+^-ATPase (FPKM = 60163.70), the alpha subunit of the F1 complex (FPKM = 23823.70), subunit C of the F_0_/V_0_ complex (FPKM = 3863.77) and subunits C and D of the V_1_ complex of the vacuolar H^+^-ATPase with FPKM values of 1448.06 and 1416.50 respectively (Additional file 8: Table S2).

**Fig. 4:**
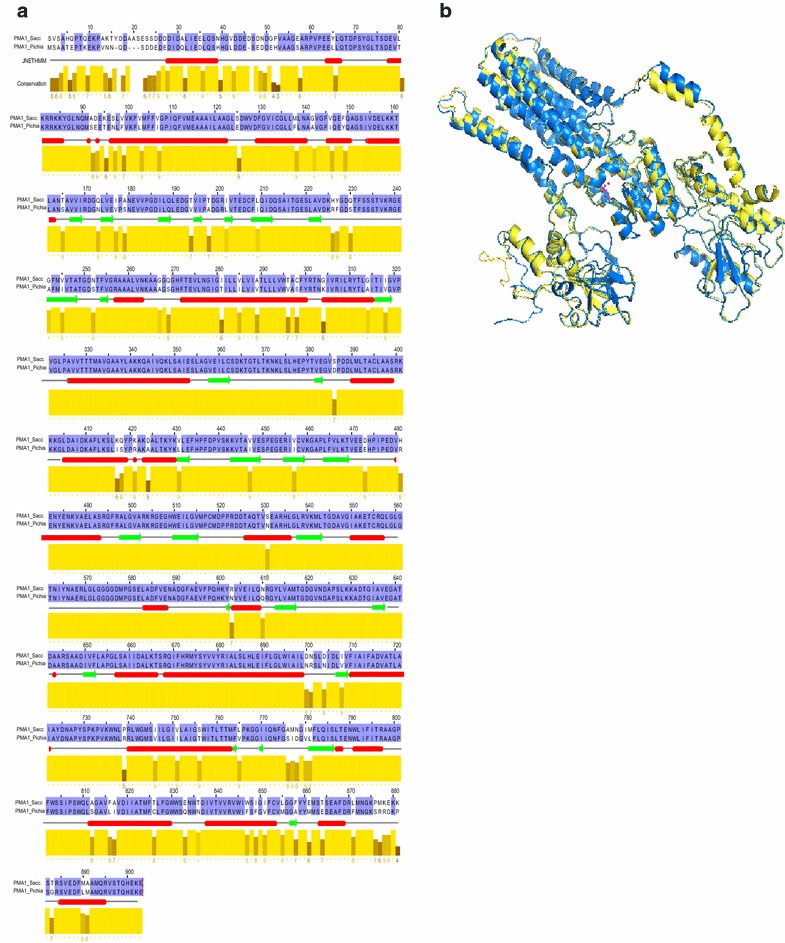
Sequence and structural comparison of the Pma1p orthologs in *S. cerevisiae* and *P. anomala*. The *P. anomala* plasma membrane H^+^-ATPase (PMA1_*Pichia*) and the *S. cerevisiae* plasma membrane H^+^-ATPase (PMA1_*Sacc*) are highly conserved with 84 % sequence identity (**a**). Predicted secondary structure is depicted below the alignment (*red* represents the α-helix and the β-sheet is shown in *green*). The conservation scores are plotted as *bar plot* below the secondary structure prediction line for each alignment column. The predicted 3D structures are compared and displayed as *yellow* for PMA1_*Pichia* and* blue* for PMA1_*Sacc* (**b**). The statistics of the predicted structures and corresponding comparison are shown in the Additional file 10: Table S3)

While it seems that the high expression of the H^+^-ATPase pumps (proton pump) in the plasma and vacuole membrane plays a critical role in pH homeostasis in *P. anomala*, these pumps are highly conserved in other fungi and there are reports that they are activated in *S. cerevisiae* under different stress conditions [20, 21]. Next, in order to identify any unique features of the *P. anomala* plasma membrane H^+^-ATPase that might play a role in making *P. anomala* more resistant than *S. cerevisiae* to low pH tolerance, the amino acid sequence of the plasma membrane H^+^-ATPase from *P. anomala* was aligned with its ortholog in *S. cerevisiae* (Pma1p) using the MUSCLE [22] and Jalview alignment programs [23]. The alignment indicated that even though the *S. cerevisiae* proton pump had 16 amino acids more than that of *P. anomala* both shared a number of highly conserved amino acid residues and both pumps shared 84 % identity (Fig. 4a). We also generated a structural model of the two proton pumps and a comparison of the structural models further confirmed the high level of similarity (Fig. 4b). Like the *S. cerevisiae* proton pump, the *P. anomala* proton pump had two conserved Pfam domain functions—E1/E2 ATPase and, the hydrolase domain. The E1/E2 ATPase and hydrolase domains of the *P. anomala* proton pump had a percentage identity of 85.65 and 90.91 respectively with the *S. cerevisiae* pump. Therefore, there is not any significant difference at the amino acid sequence level reflecting a high level of structural and perhaps, functional similarity between the two H^+^-ATPases (Additional file 9: Table S3).

### Oxidative phosphorylation and the electron transport chain

Among the highly expressed genes, the following GO terms were also over-represented—oxidoreductase activity (GO:0016491), ubiquinol cytochrome-c activity (GO:0008121), electron transfer activity (GO:0009055) and succinate dehydrogenase activity (GO:0000104) alongside the terms mapped to the H^+^-ATPase activity (Fig. 3b). The high expression of genes associated with these GO terms reflect an active production of ATP through mitochondrial respiration in the *P.anomala* strain. In addition, some genes were mapped to the protein serine/threonine kinase activity GO term (GO:0004674) that might play a role in phosphorylation of specific serine and threonine residues in the H^+^-ATPase or associated proteins [24, 25].

We also performed a homology search to describe the *P. anomala* electron transport (respiratory) chain based on the *S. cerevisiae* oxidative phosphorylation chain. Using the KEGG database [26], we gathered all the protein components of *S. cerevisiae* electron transport chain and used them as queries to perform a blastp in JGI genome browser for *P.anomala* (http://www.genome.jgi.doe.gov/pages/blast-query.jsf?db=Wican1). Indeed, all the homologs of the *S. cerevisiae* respiratory chain were also found in *P. anomala* with an average sequence identity of 62.38 % (Additional file 10: Table S4). Several of the homologs were highly expressed in the *P. anomala* strain we isolated (Additional file 8: Table S2). It is worth noting, however, that unlike most yeasts, *S. cerevisiae* has several peripheral membrane NADH dehydrogenases instead of Complex I in the respiratory chain [27]. Therefore, in order to determine the presence of Complex I in *P. anomala*, we used the Complex I subunits from *Pichia pastoris* available in the KEGG database [26] for a blastp search against *P. anomala*. Interestingly, we found 17 *P. pastoris* Complex I subunits present in the *P. anomala* genome with an average identity of 61.81 % (Additional file 10: Table S4). These subunits were not found in the *S. cerevisiae* respiratory chain. To determine if these subunits were expressed, we checked their FPKM values in our RNA-seq data and found that 12 of the identified subunits were highly expressed in the *P. anomala* strain having FPKM values in the range of 34–180. The most highly expressed subunits were the acyl carrier protein (ACP) of Complex I, NADH:ubiquinone oxidoreductase, F subunit and NDUFA5/B13 subunit with an average FPKM of 160 which confirms the active presence of Complex I in *P. anomala* (Additional file 8: Table S2).

## Discussion

Here, we studied low pH stress responses in an acid resistant yeast, *P. anomala* and have demonstrated how whole genome sequencing and RNA-seq can be used to characterise low pH stress responses in a non-model yeast strain. Yeasts have become a more preferred microbial cell factory for different industrial applications. Currently, *S. cerevisiae* which has been very well-characterised is mostly used as the main workhorse for several biological processes. However, the industrial use of this strain is challenged by its limited ability to deal with different stress conditions imposed during industrial fermentations. One of these stresses is low pH or acid stress. Apart from improving the tolerance of *S. cerevisiae* to stress, an alternative approach will be to isolate and characterise novel yeast strains that have a natural resistance to stresses such as acid stress [28] in order to identify novel tolerance mechanisms in these strains.

One well-established mechanism by which cells deal with acid stress resulting from intracellular accumulation of protons is by pumping the excess protons out of the cytoplasm into the extracellular environment or into the vacuole [29]. In yeasts and other micro-organisms, this process is mainly carried out by H^+^-ATPases (proton pumps). Several components of ATPases were highly expressed with the membrane H^+^-ATPase being the most highly expressed gene as shown in the transcriptomic data presented in this work. Plasma membrane H^+^-ATPases belong to a large family of molecular pumps involved in ion transport across the membrane [30] and have already been shown to be activated in yeast when the external environment is acidified [31]. The H^+^-ATPases pump out excess protons which diffuse into the cytoplasm in an ATP dependent process thus maintaining the internal pH of the cell and has been shown to be activated during acid stress showing its relevance in pH control [21]. Our data confirm previous work by Fredlund et al. [8] who first reported the unique low pH resistance phenotype of *P. anomala*. To the best of our knowledge, the *P. anomala* H^+^-ATPase has not been previously reported to play a role in acid stress tolerance since prior to this present study there has not been any genetic and transcriptomic study on acid stress responses displayed by *P. anomala*. Subunits of the vacuolar H^+^-ATPase (V-ATPase) were also highly expressed even though the level of expression as measured by the FPKM values was not as high as the plasma membrane H^+^-ATPase. The V-ATPase works in tandem with the plasma membrane H^+^-ATPase to de-acidify the cytosol by pumping excess protons from the cytosol into the vacuole [32]. Therefore, the expression of *P. anomala* V-ATPase in this study shows its contribution to increasing low pH tolerance in the yeast. The high expression of H^+^-ATPases provides an obvious explanation to acid stress tolerance. However, a closer look at the structure of the *P. anomala* plasma membrane H^+^-ATPase shows a high level of homology to the *S. cerevisiae* plasma membrane H^+^-ATPase (Pma1p) even though *P. anomala* is more resistant to low pH than *S. cerevisiae*. Therefore, increased expression of the *P. anomala* H^+^-ATPases alone cannot explain the basis of its high resistance to low pH.

Expulsion of protons by H^+^-ATPases is an energy intensive process which drains the cell of most of its intracellular ATP [33]. Interestingly, we also observed a high expression of succinate dehydrogenase and several oxidoreductases in our work, which was an indication of an active energy generation mechanism by the cell through the respiratory chain and may provide a further explanation to the low pH tolerance of *P. anomala*. Succinate dehydrogenase is a key component and a functional member of the electron transport chain [34] which is closely linked to the oxidative phosphorylation pathway where the oxidoreductases mediate the conversion of mitochondrial ADP to ATP [34]. In effect, ATP production by the mitochondria will be increased by the electron transport chain and the ATP produced is supplied to the cell to meet the ATP requirements to exclude protons out of the cell by the plasma membrane H^+^-ATPase. There is usually a high energy demand by microbial cells under environmental stress [35] and it has also been mentioned by de Kok et al. [36] that the H^+^/ATP stoichiometry of the plasma membrane H^+^-ATPase determines the ATP requirement of the cell for maintenance and H^+^ ion homeostasis. Therefore, increasing ATP supply within the cell is beneficial in alleviating the stress and this has indeed been reported by Zhou et al. [35] where acid tolerance of *Candida glabrata* was improved by supplying the cell with excess amounts of ATP. The structure of the respiratory chain in *P. anomala* in comparison with that of *S. cerevisiae* further highlights the ability of *P. anomala* to efficiently generate energy from respiration. Although core proteins were conserved in the respiratory chain of both yeasts, *S. cerevisiae* lacks most subunits of Complex I as a result of an evolutionary adaptation to fermentative anaerobic growth [37]. Conversely, the presence of Complex I in *P. anomala* will enable the yeast to produce substantial amounts of energy predominantly through the electron transport chain since this Complex I generates 40 % of the transmembrane electrochemical gradient needed to synthesise ATP from ADP in the mitochondria [38].

In the presence of high extracellular amounts of glucose, *S. cerevisiae*, ferments the substrate to yield ethanol. Even though ATP is produced in this pathway, the yield is not high enough to meet the energy demand of the cell under acid stress unlike in *P. anomala*, which respires actively to generate much more ATP as shown in our study. Similarly, Chen et al. [39] observed a down-regulation of genes involved in the TCA cycle and in respiratory metabolism during acid stress in *S. cerevisiae* even though pyruvate decarboxylase activity was increased signifying a direction of carbon flux toward ethanol production while respiration was turned off. Furthermore, other reports characterising the effect of low pH on *S. cerevisiae* at both genomic and transcriptomic levels do not show an activation of mitochondrial ATP production. Instead, other mechanisms including activation of the protein kinase pathway which plays a role in maintaining cell shape and integrity [40] were identified. Also, upregulation of metal metabolism has been shown to be important in acid stress resistance in *S. cerevisiae* [10]. Under conditions of acid stress, priority is given to dealing with proton accumulation in the cell by extruding the excess protons into the extracellular environment through the plasma membrane ATPase. Data presented in this study show that even though both *S. cerevisiae* and *P. anomala* consume glucose at the same rate under low pH conditions, *S. cerevisiae* has a lower biomass yield on glucose but a higher ethanol and glycerol production rate compared to *P. anomala*. This implies that *P. anomala*, through respiration resulting from its Crabtree negative nature, is able to generate a larger pool of ATP to deal with the stress and for maintenance (evidenced from the high biomass yield) whereas *S. cerevisiae* uses its limited supply of ATP (from glycolysis and fermentation) to provide the chemical energy needed by the plasma membrane ATPase to transport the excess protons out of the cell at the expense of cell maintenance reflected in a low biomass yield.

In summary, although the plasma membrane H^+^-ATPase of both *P. anomala* and *S. cerevisiae* show a high level of homology and both strains consume glucose at the same rate during acid stress, *P. anomala* proved to be more resistant to low pH than *S. cerevisiae*. Our findings suggest that the mere presence of a proton pump does not necessarily lead to tolerance to acid stress but an improved supply of chemical energy (ATP, in this case) is needed to power the proton pump. Yet, other factors that can remarkably influence the activity of Pma1p include the lipid composition of the membrane [41] and the regulatory mechanism underlying Pma1p expression. Thus, in vivo validation of Pma1p activity is needed to back transcriptional data. While this study characterises tolerance of *P. anomala* to inorganic acid stress, it will still be interesting to investigate transcriptional responses of this yeast to stress posed by different organic acids since tolerance to low pH does not necessarily imply tolerance to organic acids at low pH particularly because apart from the stress stemming from the low pH environment, accumulation of the anion form of an organic acid in the cytoplasm will also be toxic to the cell.

## Methods

### Isolation and identification of the yeast

Five strains of yeasts were isolated from different low pH (pH 3.0) cultures and were identified as *P. anomala* by a PCR amplification of the Internal Transcribed Sequence (ITS) region of the yeast genome which includes the conserved sequences of 5.8S and 28S ribosomal DNA [42]. The ITS 1 (TCCGTAGGTGAACCTGCGG) and ITS 4 (TCCTCCGCTTATTGATATGC) primers [42] were used for the PCR amplification. The yeast genomic DNA was extracted using the LiOAc-SDS method as described by Looke et al. [43]. The PCR products were sequenced by Eurofins Genomics (Ebersberg, Germany) and the species were identified through a BLASTN search (http://www.blast.ncbi.nlm.nih.gov/Blast.cgi).

### Growth rate determination

Pure cultures of the yeast strains were obtained by isolating individual colonies on solid YPD (10 g/L yeast extract, 20 g/L peptone, 22 g/L glucose) media plates. Fresh minimal medium [7.5 g/L(NH_4_)_2_SO_4_, 14.4 g/L KH_2_PO_4_, 0.5 g/L MgSO_4_·7H_2_O, 20 g/L glucose, 1 mL/L vitamin solution and 1 mL/L trace elements solution) with pH adjusted to 3.0 with 1 M HCl was inoculated with overnight cultures of the strains to an initial OD_600_ of 0.05. The cultures were incubated with shaking (200 rpm) at 30 °C and the cell density (OD_600_) of the cultures were measured every 2 h with a Thermo Scientific Genesys20 spectrophotometer (Waltham, USA). In a parallel experiment, the growth rate of *S*. *cerevisiae* CEN.PK 113-7D was obtained for comparison with the *P.anomala* strains under the same conditions. The growth rate was determined from the slope obtained from a semi-logarithmic plot of OD_600_ against time during the exponential phase of the cultures. The top-performing *P. anomala* strain out of the five isolated strains was used for the subsequent experiments and deposited in our strain collection as *P. anomala* EF01.

### Quantification of glucose and fermentation metabolites

Aliquots of the supernatants of the *S. cerevisiae* cell cultures and that of the top-performing *P. anomala* were taken every 2 h, kept at 4 °C and were used to determine the concentration of substrates and other fermentation metabolites by HPLC analysis. Using an RI and UV detectors, an Aminex HPX-87H column (Biorad, Irvine, CA, USA) was used to separate and quantify glucose, ethanol and glycerol in the supernatants. The column was eluted with 5 mM H_2_SO_4_ eluent at a flow rate of 0.6 mL/min.

### Sample and library preparation

Genomic DNA was extracted from top-performing *P. anomala* strain using the Qiagen Gentra Puregene kit (Hilden, Germany). To obtain the total RNA of the *P. anomala* strain, 10 mL cultures were grown for ~8 h in minimal medium (pH 3.0) until they reached the mid-log growth phase (OD = 1). The total RNA was then isolated from these cultures in three biological replicates using the Qiagen RNAeasy Mini Kit (Hilden, Germany).

### Sequencing and data preprocessing

The Illumina sequencing platform (HiSeq 2000, 100-bp paired-end) at the Theragen Bio Institute, South Korea was used to sequence the genomic DNA libraries while the Multiplex Illumina HiSeq High Output mode (2 × 100 bp paired-end) was used to sequence the RNA libraries. The sequences were pre-processed by Trimmomatic [44] in order to remove adaptors, leading low quality or N bases (below quality 3), quality trimming (4-base wide sliding window and quality cut off of 15), and removing reads below 36 bases long.

### De novo assembly and gene catalog assessment

For the de novo assembly we first ran Velvet [16] using VelvetOptimizer with the following parameters: –exp_cov auto (automatic calculation of expected coverage), –scaffolding (scaffolding of contigs with paired-end reads) and –min_- contig_lgth 200 (mimimun contig length = 200). The optimal k-mer length was determined by adjusting the k-mer length from 31 to 75 bp in 4-bp increments and using the k-mer for which the N50 and the maximum contig length reached the highest value (Additional file 3: Table S1). The resulting contigs were then re-assembled with CAP3 v10/15/07 using standard parameters. To compare, we also ran ABySS [14] and SPAdes [15]. For the ABySS we used the optimized suggested k-mer size of 37 based on work done by Haridas et al. [45] while keeping the other parameters as default. For the SPAdes we used the k-size range between k21 and k65 with the increment size of 4. To check if the yeast was already sequenced we blasted the longest scaffolds against JGI fungi sequenced genome databases. All the scaffolds were matched with average identity score of 98 % to the *Wickerhamomyces anomalus* NRRL Y-366-8, also known as *P. anomala* and *Hansenula anomala* in the JGI with the genome assembly size of 14.15 Mbp and 6423 gene models. For the rest of analysis we used *W. anomalus* NRRL Y-366-8 as our genome of reference. To compare the quality of our de novo assemblies we used the QUAST program [17] with *W. anomalus* as the reference genome and chose –*gene*-*finder* and –*eukaryote* parameters to predict the genes.

Among the assemblers that we used ABySS output had the highest genome coverage (>80 %) and the coverage of the Velvet-assembled genome was less than 50 %.

### RNA-seq analysis

For the RNA-seq analysis we used the Tuxedo pipeline (Additional file 11: Figure S7) [18]. However, we used *Cuffnorm* instead of *Cuffdiff* to count the reads (new extension of Cufflinks) as the former normalized the read counts to the sample librAdditionalry size.

### Go enrichment analysis

As *P. anomala* is not supported by an annotation package for GO enrichment analysis of the each of the four gene categories we used the GOstats and Category R packages which are specifically designed for hypergeometric testing with unsupported model organisms [46]. Therefore, we created a custom data structure of the GO ontologies and then each selected gene category was used as input to perform the hypergeometric test. The output of the GOstats was visualized using REVIGO [19].

### Sequence and structural bioinformatics analysis

The orthologs of the *P. anomala* plasma membrane H^+^-ATPase were detected using the BLASTp program [47]. Jalview was used for the pairwise alignment and visualization [23]. The secondary structures of the *S. cerevisiae* and *P. anomala* H^+^-ATPases were predicted as described in [48, 49]. For the prediction and alignment of the structures we used the RaptorX server [50].

## Additional files

**Additional file 1: Figure S1.** Gel image showing the PCR product obtained from the amplification of the ITS region of *S. cerevisiae *(Lane 1) and *P. anomala* (Lane 2). Lane M represents a 1 kb DNA ladder (Thermo Scientific, Vilnius – Lithuania).
**Additional file 2: Figure S2.** Growth rate of five *P. anomala* strains grown in low pH (pH 3.0) minimal medium at 30 °C.
**Additional file 3: Table S1.** Comparison of the three de novo assemblers used in this study.
**Additional file 4: Figure S3.** The expression cutoff based on log10 FPKM values to categorize gene expression level.
**Additional file 5: Figure S4.** GO enrichment analysis of the genes with low-to-medium expression level.
**Additional file 6: Figure S5.** GO enrichment analysis of the genes with medium-to-high expression level.
**Additional file 7: Figure S6.** GO enrichment analysis of the genes with enriched expression level.
**Additional file 8: Table S2.** Expression levels of genes involved in respiration and proton efflux.
**Additional file 9: Table S3.** Statistics for the structural comparison from RaptorX.
**Additional file 10: Table S4.** Components of the respiratory chain in *P. anomala* that have homologs in *S. cerevisiae* obtained from a protein BLAST.
**Additional file 11: Figure S7.** RNA-seq analysis pipeline using Tuxedo.
