# Supplementary figures and images for: RNA-seq analysis of Pichia anomala reveals important mechanisms required for survival at low pH
Source: Microb Cell Fact. 2015 Sep 16;14:143. doi: 10.1186/s12934-015-0331-4 (PMC4574170; doi:10.1186/s12934-015-0331-4)

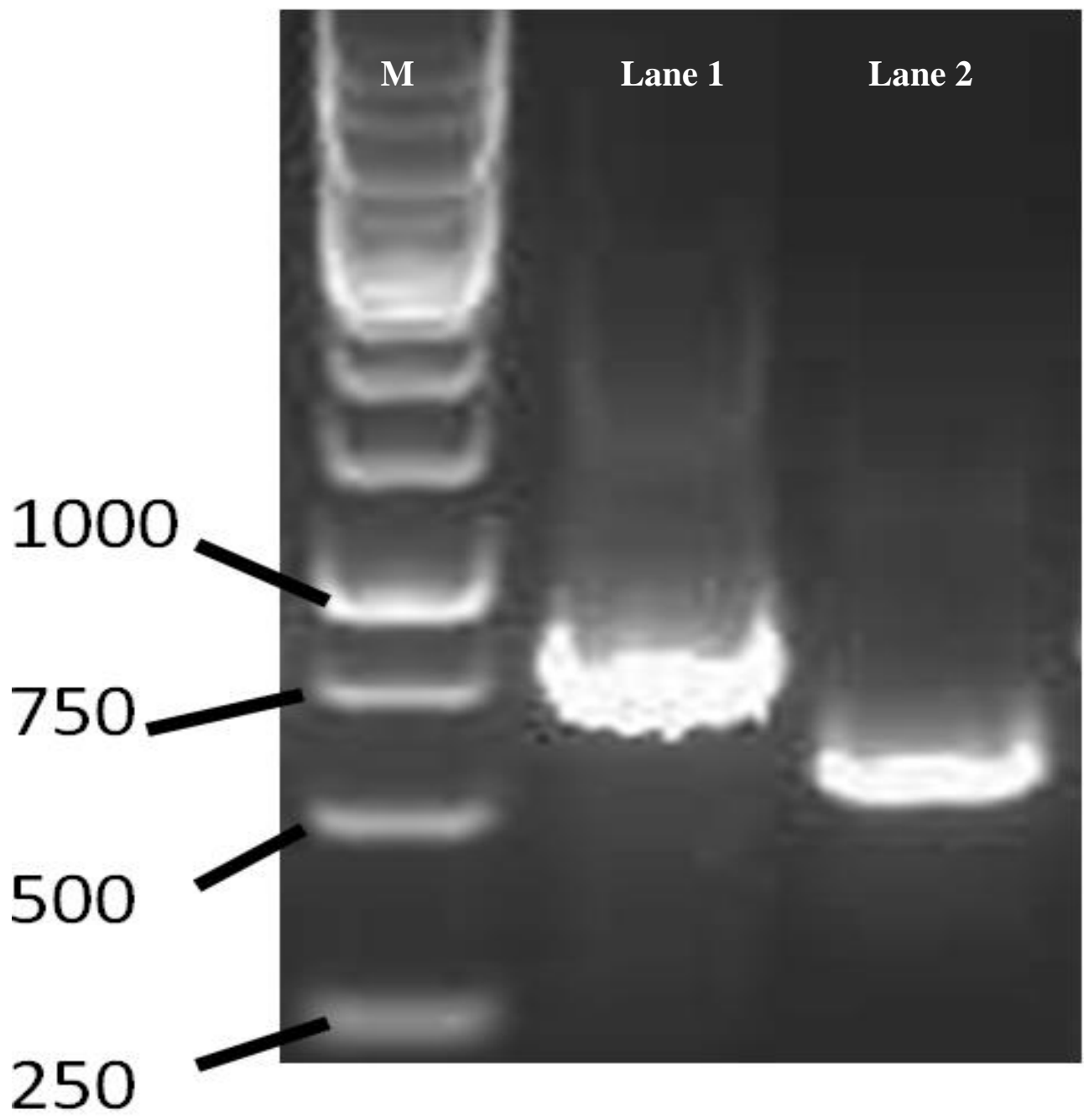

Supplement: Supplementary file 1 — Additional file 1: Figure S1. Gel image showing the PCR product obtained from the amplification of the ITS region of S. cerevisiae (Lane 1) and P. anomala (Lane 2). Lane M represents a 1 kb DNA ladder (Thermo Scientific, Vilnius – Lithuania). [file 12934_2015_331_MOESM1_ESM.pdf]

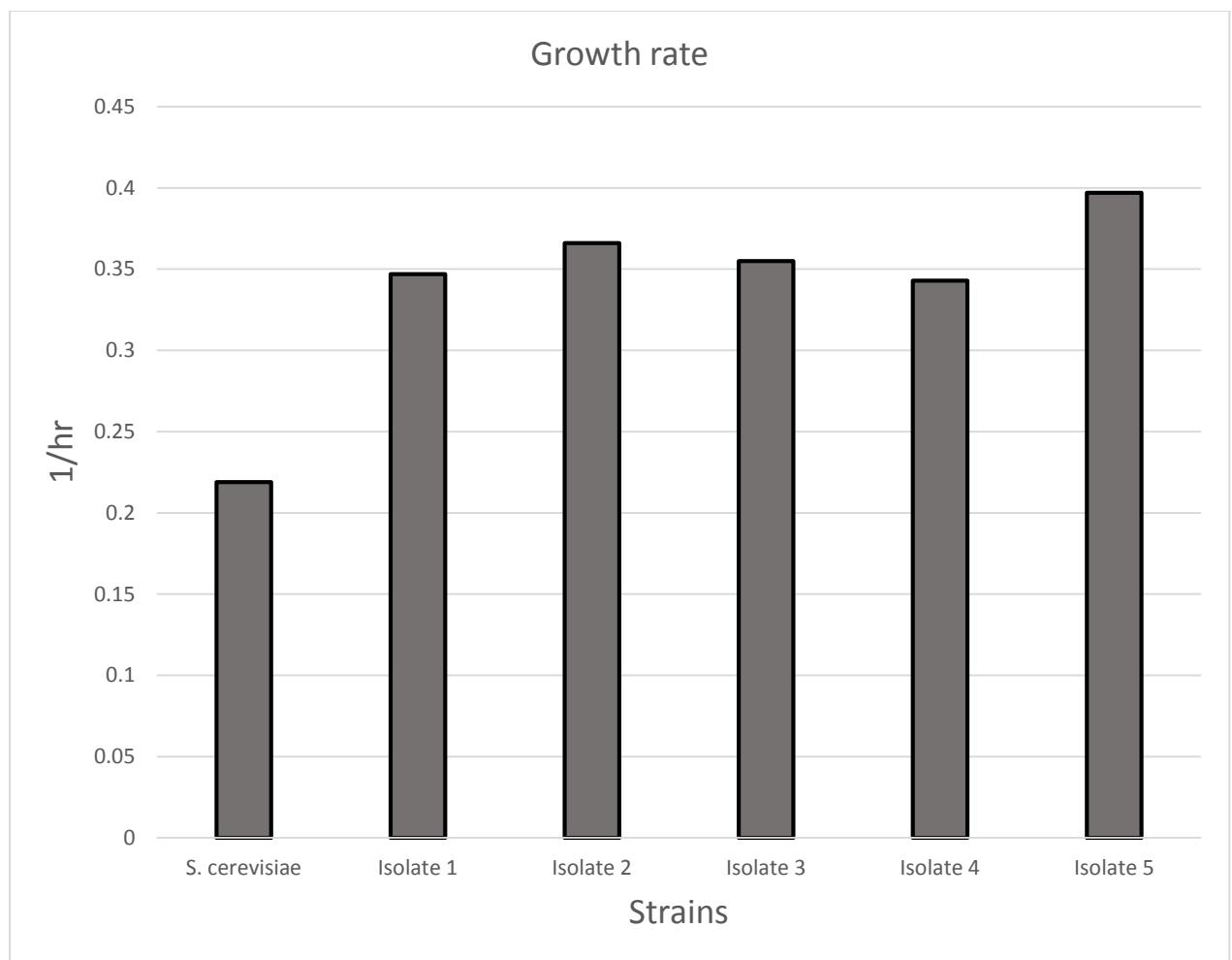

Supplement: Supplementary file 2 — Additional file 2: Figure S2. Growth rate of five P. anomala strains grown in low pH (pH 3.0) minimal medium at 30 °C. [file 12934_2015_331_MOESM2_ESM.pdf]

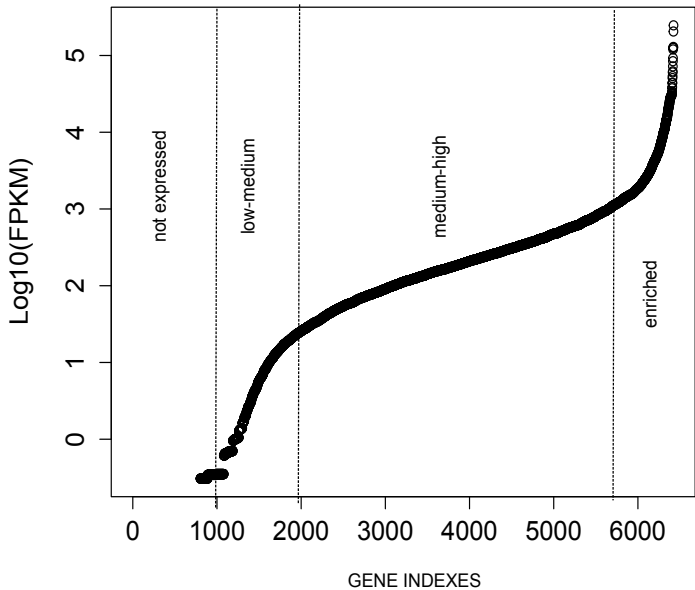

Supplement: Supplementary file 4 — Additional file 4: Figure S3. The expression cutoff based on log10 FPKM values to categorize gene expression level. [file 12934_2015_331_MOESM4_ESM.pdf]

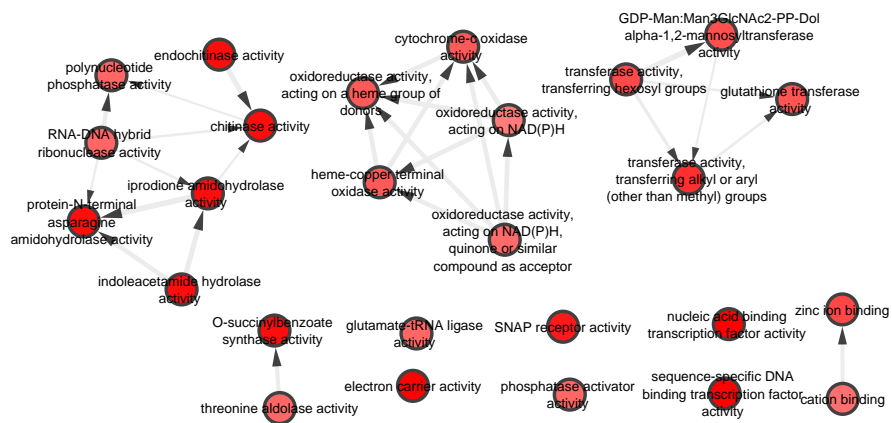

Supplement: Supplementary file 5 — Additional file 5: Figure S4. GO enrichment analysis of the genes with low-to-medium expression level. [file 12934_2015_331_MOESM5_ESM.pdf]

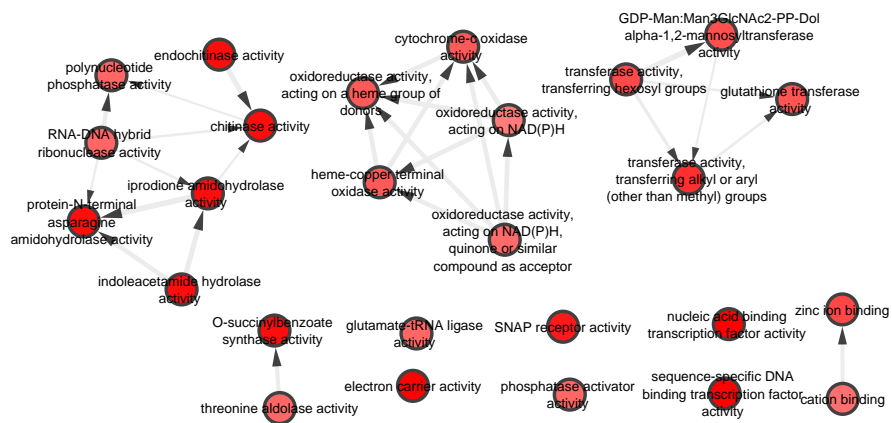

Supplement: Supplementary file 6 — Additional file 6: Figure S5. GO enrichment analysis of the genes with medium-to-high expression level. [file 12934_2015_331_MOESM6_ESM.pdf]

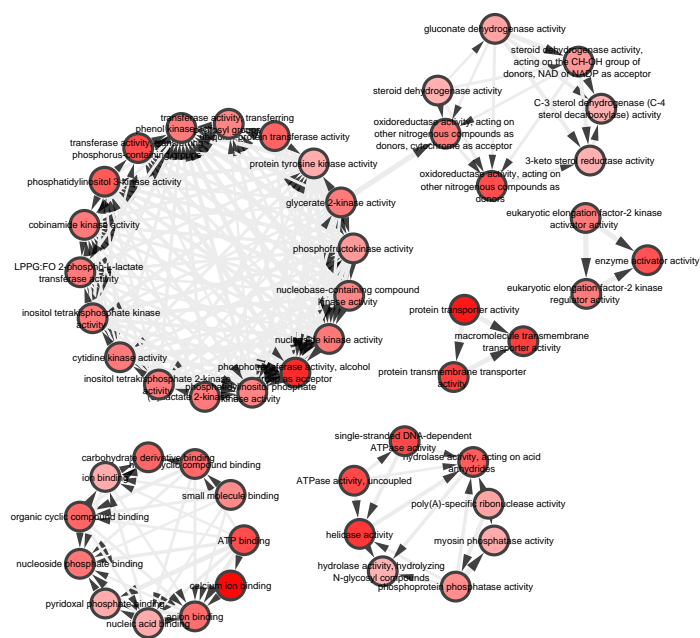

Supplement: Supplementary file 7 — Additional file 7: Figure S6. GO enrichment analysis of the genes with enriched expression level. [file 12934_2015_331_MOESM7_ESM.pdf]

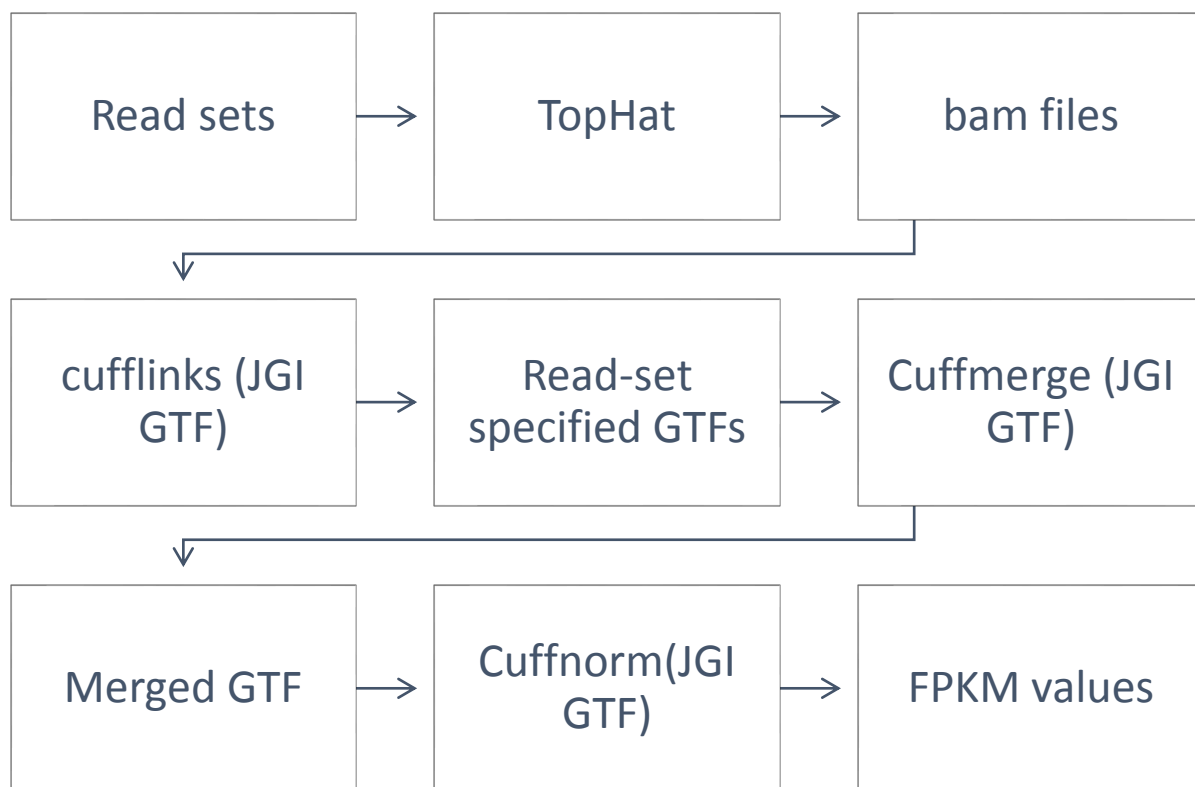

Supplement: Supplementary file 11 — Additional file 11: Figure S7. RNA-seq analysis pipeline using Tuxedo. [file 12934_2015_331_MOESM11_ESM.pdf]
